# Examination of *Salmonella* prevalence and slaughter findings in pigs based on rye feeding, coarser feed structure and vaccination against *Lawsonia intracellularis* under field conditions

**DOI:** 10.1016/j.vas.2026.100693

**Published:** 2026-05-11

**Authors:** Jens Gerrit Lindhaus, Karl Rohn, Christian Visscher, Clara Berenike Arnold

**Affiliations:** a Institute for Animal Nutrition, University of Veterinary Medicine Hannover, Foundation, Bischofsholer Damm 15, D-30173 Hanover, Germany; b Institute of Biometry, Epidemiology and Information Processing, University of Veterinary Medicine Hannover, Foundation, Bünteweg 2, D-30559 Hanover, Germany

**Keywords:** Gut health, Zoonotic agent, Dietary concepts, *Salmonella*, Rye, *Lawsonia intracellularis*, Pig, Vaccination

## Abstract

*Salmonella* remains a food safety concern through pork. This field study assessed the impact of high rye diets and *Lawsonia intracellularis* (LI) vaccination on *Salmonella* seroprevalence and pathological findings at slaughter in fattening pigs. Six commercial farms participated in a three-phase trial with varying rye levels and optional immunoprophylaxis against LI.

From Phase 1 to 3, rye content increased progressively (pre-, mid-, finishing phase). In Phase 1 (P1), the proportion increased from 5% to 20% to 40%. Phase 2 (P2) showed a higher inclusion level, with ≥30 to ≥40 to 60–70%. In Phase 3 (P3), rye inclusion increased from 30% to 50%, also a LI vaccination was introduced.

*Salmonella* serology was available for four farms across all phases; slaughter data (pathological findings) for six farms in Phases 2–3.

Increasing dietary rye levels (P1 to P2; P1 to P3) significantly lowered *Salmonella* optical density percentage (OD%), especially on farms with high baseline contamination (Farm 1: P1 13.96%, P2 2.78% P3 2.88%). In P3, a reduction in slaughter findings, particularly lung and pleural abnormalities (Farm 1–4: P2 20.54%, P3 15.19%), was observed following LI vaccination. Highest prevalence of pathological findings (lung and pleura: Farm 6: P2 60.31%, P3 66.15%) was noted without any measures.

These findings highlight the value of gut health management through nutrition. High dietary rye levels appear to stabilize microbiota, strengthen the intestinal barrier and reduce *Salmonella* antibodies. Combining tailored feeding regimes with preventive health protocols may enhance pig resilience and improve food safety in modern swine production.

## Highlights

• Field study in fattening pigs evaluating feeding measures and vaccination.
• Valuable insights gained from slaughter data – no experimentation on live animals.
• Rye in high shares can help tackle Salmonella antibody counts in fattening pigs.
• Combined measures improve overall health.

## 1 Introduction

Salmonellosis is a very well-known zoonosis worldwide and is responsible for a large proportion of foodborne illnesses (EFSA, 2024; Robert Koch Institute, 2025). In particular, the serovars *Salmonella typhimurium* and *Salmonella enteritidis* are responsible for the second most common bacterial foodborne illness in Germany, according to the Robert Koch Institute (Robert Koch Institute, 2025). Elevated *Salmonella* prevalence can be seen daily in pigs at slaughterhouses across Europe (EFSA, 2024; Powell et al., 2016). As a result, measures such as systematic detection in slaughter pigs have become standard practice a long time ago (Justiz & Verbraucherschutz, 2007). Initial interventions such as hygiene management, monitoring programs or dietary supplements like acids have had less impact than expected, making new preventive strategies necessary (Majowicz et al., 2010).

The most commonly used cereal sources for pigs include wheat, barley, maize and triticale (Bederska-Lojewska et al., 2017) but due to the good acceptance of high amounts of rye in the feed and its high content of dietary fiber, many positive aspects of including rye into the diet for pigs were identified in the last years (Chuppava et al., 2020; Hartung et al., 2024; Lindhaus et al., 2024; McGhee et al., 2021). Especially the fraction of non-starch polysaccharides (NSP) and the feed structure have become interesting for pig feeding, because of the bacteriostatic or bacteriolytic effect after the fermentation of NSP with production of butyrate in the large intestine (Bach Knudsen et al., 2019). Rye contains higher amounts of soluble dietary fiber than other cereal grains because of its higher concentration of soluble NSP (Rodehutscord et al., 2016). Arabinoxylans and fructans form a large proportion of the NSP and are mainly fermented in the large intestine, which leads to the formation of short chain fatty acids (SCFA), especially butyrate and propionate, in the caecum and colon by beneficial gut microbes (Glitsø et al., 1998; Hartung et al., 2025; Wilke, 2020). This results in increased intestinal villi height and in improved overall gut health (Biagi et al., 2007; Correia et al., 2024). Improved gut health can also lead to improved overall health (Hankel et al., 2022). Butyrate has long been used against *Salmonella* infections and contributes to intestinal health (Van Immerseel et al., 2006; Visscher et al., 2009). Favoring a fermentation pattern with elevated production of butyrate and propionate could reduce invasive *Salmonella* infections in animals (Gantois et al., 2006; Glitsø et al., 1998; Van Immerseel et al., 2006; Visscher et al., 2009). A reduced pH value in the intestine reduces the ability of *Salmonella* proteins to penetrate the intestinal wall and into the intestinal epithelial cells, thereby reducing the exposure of pigs to *Salmonella*. (Boyen et al., 2008; Lawhon et al., 2002; Van Immerseel et al., 2006;). In addition, a reduced pH value leads to reduced skatole formation, which can in turn reduce boar taint (Wesoly et al., 2012). Previous studies have shown that *Lawsonia intracellularis* (LI) has similar infection dynamics as *Salmonella* spp. and that pigs are often infected with both pathogens simultaneously, which is characterized by additive inflammatory responses and disruption of the microbiome (Brandt et al., 2010; Moller et al., 1998; Stege et al., 2000;). In addition, infection with LI has been shown to promote persistent shedding of *S. typhimurium* and, in the case of *S. enteritidis* to contribute to pigs becoming latent carriers (Kranker et al., 2003). These results led to the hypothesis that pigs infected with LI have a higher risk of contributing to zoonotic *Salmonella* infections in humans (Patterson et al., 2016). In response to this problem, studies are looking at whether vaccination against LI can reduce *Salmonella* prevalence in pigs. Visscher et al. (2018) proved that under experimental conditions, vaccination against LI in pigs could significantly reduce the excretion of *Salmonella*. In addition, vaccination with attenuated live LI causes a reduction in the elevation of *Salmonella* in the acute infection and improves the gut microbiome (Borewicz et al., 2015; Leite et al., 2018; Meschede et al., 2021; Visscher et al., 2018).

The aim of this field study was to show, whether pigs whose *Salmonella* antibody prevalences had already been reduced by an adapted diet with a rye content of up to 70% and a coarse feed structure could achieve further reductions in *Salmonella* prevalence and whether vaccination against LI also has an effect on other health parameters (pathological findings) that are routinely monitored at the slaughterhouse. It was hypothesized, that the preventive intervention in feeding and vaccination could reduce antibody prevalence and, consequently, *Salmonella* infections as well as improve overall health and resilience of the animals.

## 2 Materials and methods

Based on data from >175 pig farms, four farms were selected that were comparable in piglet origin, management conditions, diet formulation, and vaccination scheme, but differed in location. These farms were evaluated longitudinally for *Salmonella* prevalence and slaughter findings under a defined nutritional concept and vaccination program. Two further farms were included as comparators, differing by the absence of dietary adjustment and by vaccination status.

The decision to analyze Farms 1–4 collectively in Table 3 is methodologically justified, given their uniform dietary and health management protocols. Their comparison with Farm 5 (no rye) and Farm 6 (no rye, no vaccination) allows a targeted evaluation of the individual and combined effects of dietary measures and immunoprophylaxis.

The basis for data collection for *Salmonella* antibodies was done in the same procedure as described in Lindhaus et al., 2024. The data collection was conducted in accordance with the Swine *Salmonella* Ordinance and aligned with German law (Justiz & Verbraucherschutz, 2007). The serological testing for *Salmonella* was performed during the slaughter process, and no interventions were carried out on live animals, as defined by the German Animal Welfare Act (Justiz & Verbraucherschutz, 2007). The purpose of this study was to evaluate the influence of feeding strategies in combination with vaccinations on *Salmonella* serology and findings of pathologic organ alterations during slaughter in pig fattening. To ensure proper nutrition, only complete feed was used throughout the study, preventing any nutrient deficiencies in the animals (Lindhaus et al., 2024). The study was divided into three phases: P1 (2017–2019), P2 (2020), and P3 (2021–2022). Adjustments to the feeding concept with regard to rye were implemented in P1, P2, and P3. Vaccination only took place in P3. *Salmonella* data were available for all three phases (P1, P2, P3), although slaughter findings were only available for P2 and P3. All participating farms volunteered for the study and adjusted their feeding concepts accordingly. The farms kindly provided us with all the data available to them. We were unable to include any more data than presented here.

The results were analyzed in two parts. First, four pig farms (*n* = 4) were analyzed with regard to the prevalence of *Salmonella* antibodies during adapted feeding, characterized by an increased rye inclusion (P1: 5%, 20%, 40%; P2: ≥30, ≥40%, 60–70%; P3: 30%, 50%) and a coarser feed structure, in combination with vaccination (LI). These effects were assessed across three phases: P1 (2017–2019), P2 (2020), and P3 (2021–2022), whereby vaccination only took place in P3.

Second, six farms (*n* = 6) were evaluated for pathological findings recorded at slaughter, including abnormal lesions of the heart, liver, lungs and pleura, odor as well as ear and tail alterations (Table 1). For Farm 1–4, slaughter findings data for P2 and P3 were available in addition to the *Salmonella* data. For comparison Farm 5 was included without feed adjustment but with vaccination, whereas Farm 6 was included without feed adjustment or vaccination. Slaughter data for P2 and P3 were available for both farms.

**Table 1:** Classification of slaughter findings (odor, lung and pleura, heart, liver, ear, tail) in pigs on the slaughterhouse.

Organ	Lesions	Key	Description
Odor	no odor	0	no significant findings
	odor	1	boar taint
Lung and pleura	without lesions	−1	no significant findings
	10%	0	mild lesions
	10% - 30%	1	moderate lesions
	30%	2	severe lesions
Heart	without lesions	0	no significant findings
	lesions	1	findings
Liver	without milkspots	0	no significant findings
	milkspots	1	showing Milkspots
Ear	lesions	0	no significant findings
	without lesions	1	with necrosis, inflammation
Tail	without lesions	0	no significant findings
	necrosis, inflammation	1	with necrosis, inflammation

### 2.1 Farms

All farms were located in the north-west of Germany. The farms ranged in size up to 2000 fattening places. The type of livestock consisted exclusively of conventional husbandry. The farms had an average of three fattening runs per year.

### 2.2 Animals

The pigs on the farms were conventionally housed with uniform final slaughter weights and bred under standardized procedures. Also, the pigs from farms 1–4 came from the same piglet producer. A total of 27,673 slaughter findings originating from six farms were recorded based on post-mortem examinations, with a focus on pathological changes and boar taint. These data were derived from the slaughterhouse, with an average of 48.32 pigs per delivery. On average, 1.06 findings per animal were documented, which were categorized into six groups: lung and pleura, liver, heart, odor, tail, and ear. In addition, a total of 1775 serum samples from four farms were collected across three experimental phases for the determination of *Salmonella*-specific antibodies.

### 2.3 Feeding, ration designs and vaccination

The most commonly used feeding method on the farms was liquid feeding (*n* = 5). Only one farm (*n* = 1) used a mixture of liquid and mash feeding.

In P1 (2017–2019), a specific feeding concept was defined for pre-fattening phase (28–60 kg), mid-fattening phase (60–80 kg) and finishing fattening phase (>80 kg). A maximum of 20% of the particles in the basic feed or in the mixtures were allowed to be ≤0.25 mm in size. The proportion of rye had to be 5% in the pre-fattening phase, 20% in the mid-fattening phase and 40% in the final fattening phase. In addition, the feed mixture in the final fattening phase had to contain 25% barley and ensure a minimum ratio of 0.75 g lysine to energy (1 MJ ME). A maximum of 20% of the feed particles were allowed to be ≤0.25 mm in size. This feeding concept is identical to the feeding in the previous study by Lindhaus et al., 2024 in P1. There were no vaccinations. This phase marks the baseline in the presented study.

In P2 (2020) of the study, the feeding concept was defined as follows: In the pre-fattening phase, the rye proportion had to be ≥30%, in the mid-fattening phase ≥40% and in the final fattening phase 60–70%. The proportion of barley in the final fattening phase should be 15% of the mixed feed. Particle size and lysine to energy ratio were the same as in P1. No vaccinations against LI were carried out in P2.

In P3 (2021–2022) the following feeding adjustments were made: In the pre- and mid-fattening phase, the proportion of rye was 30% and in the final fattening phase 50%. The feeding regimen was carried out according to the same characteristics as in P1 and P2; the energy and protein supply are comparable to the previous phases.

In all five farms, that used a vaccination, vaccination protocols had already been implemented at the piglet production stage. Vaccination was performed in accordance with the manufacturer`s specifications, including administration route, timing and dosage. Of these, four farms used the same vaccine, while one farm employed a different vaccination product. Farms 1 to 4 were subject to all three phases described. Farm 5 had its pigs vaccinated but did not change its feed in P2 and P3and Farm 6 did not participate in any of the mentioned feed changes or vaccinations.

### 2.4 Data collection

A total of 27,673 organ samples were collected and examined during slaughter inspection. from 6 farms, which of 1775 individual samples from four farms were analyzed to evaluate the serological status of *Salmonella*.

The pigs that were fattened under the specified nutritional and management parameters were transported to different slaughterhouses in north-west Germany once they reached market weight. All procedures described below for data collection follow the quality assurance guidelines (Qualität und Sicherheit GmbH et al., 2026), blood and meat juice samples were collected to assess *Salmonella* exposure. During slaughter, samples of the diaphragm pillar muscle were taken to obtain meat juice, which was produced by mincing the muscle tissue.

Blood samples were collected after the pigs were stunned, stabbed and slaughtered. After centrifugation in the laboratory, the plasma or serum was used for further serological testing.

The presence of *Salmonella* antibodies in blood and meat juice samples was determined using approved ELISA test kits (Herd Check; Pri-oCheck *Salmonella* 2.0; pigtype *Salmonella* Ab). These tests detect antibodies against *Salmonella* cell wall components. A small volume of blood serum or meat juice was applied to the test kit. To ensure comparability across different ELISA kits, the results were expressed as a percentage optical density (OD%) value, which was calibrated against positive and negative controls using a conversion factor. According to the Swine *Salmonella* Ordinance, a sample was considered positive if the OD% was at least 40. Pigs with OD% values above 40 were classified as *Salmonella* seropositive. Additionally, a lower cut-off value of 10% was examined in this study to evaluate the potential for reducing *Salmonella* prevalence further. All individual results were included in the statistical analysis.

The slaughter data were obtained simultaneously during the slaughter process. Veterinary technical assistants, working under supervisor and responsibility of official veterinarians in accordance with European legislation (European Commission, 2019; European Parliament & Council of the European Union, 2017), inspected the slaughtered pigs at the abattoir with regard to six parts of the carcass: Lungs and pleura, liver, heart, odor, tail and ear. Pigs that showed an abnormality were categorized as positive. This process is embedded in a regulated system with defined criteria and continuous oversight. For example, lungs with no visible changes were classified as negative; those with changes affecting up to 10% of the lung tissue were classified as mild; those with changes affecting between 10% and 30% were classified as moderate; and those with changes affecting 30% or more were classified as severe. For our study slaughter findings were already described as positive in the event of one change without categorizing in the respective organ. The data were provided using an Excel table, which showed the number of pigs slaughtered per day and their percentage of findings per subgroup (odor, lung and pleura, heart, liver, ear, tail). The subgroups were assessed for boar taint–related odor, pathological findings in the lungs, pleura, and heart indicative of infectious or structural alterations, liver lesions of infectious, parasitic, or structural origin, as well as bite injuries or inflammatory changes affecting ears and tails. Findings were not subcategorized for severity. No procedures were carried out on live animals.

### 2.5 Statistical analysis

Data was transferred to Excel Spreadsheets (Excel, version 2019, Microsoft Corporation, Redmond, Washington, USA) and analyzed using SAS® statistical software, version 9.4M7 with SAS Enterprise Guide, version 7.15 (SAS Institute Inc., Cary, North Carolina, USA). *Salmonella* findings as a function of optical density and slaughter findings were recorded as a dichotomous variable "positive/negative" for each organ. The percentage of positive findings is then used to determine the prevalence for each organ. Prevalences of *Salmonella* antibodies and slaughter findings at the abattoir (odor, lung and pleura, heart, liver, ear, tail,) in different fattening farms between three phases (P1:2017–2019; P2:2020; P3:2021–2022) were compared using the Chi2 test. P-values ≤ 0.05 were considered statistically significant.

## 3 Results

### 3.1 Salmonella serology for farms and feeding time

**Fig. 1:**
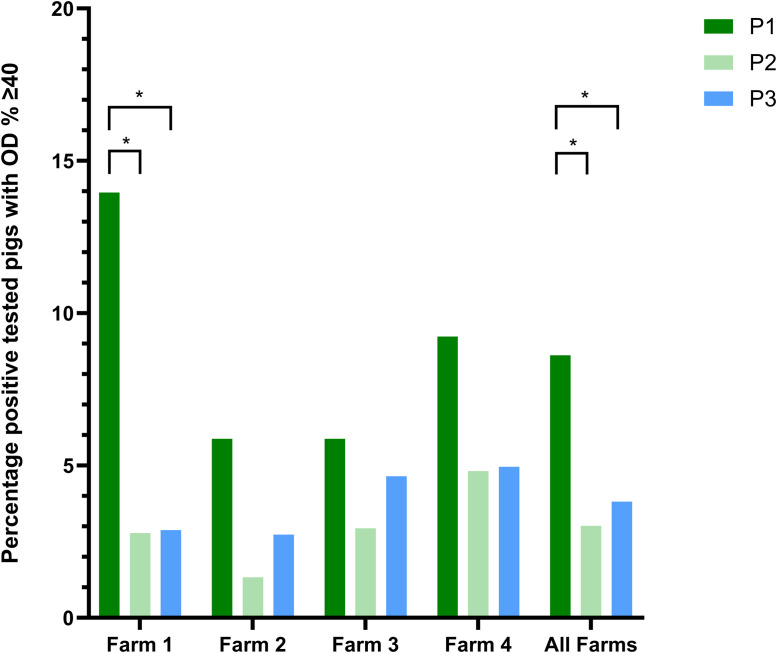
Percentage of positive tested pigs (in absolute terms) on all farms (Farm 1–4 and all together) in three different feeding and vaccination phases with an OD%≥40. Asterisks indicate significant differences between phases within a farm (*p* < 0.05).

Fig. 1 shows the percentage of positive tested pigs on Farm 1 to 4 and all farms together with an OD%≥40 in three different feeding and vaccination phases.

A significant reduction in serological positivity (OD%≥40) was observed in Farm 1, where the proportion of positive samples declined from 13.96% in P1 to 2.78% in P2 and 2.88% in P3. When data was pooled across all farms, the prevalence of positive samples was significantly higher during P1 (8.6%) compared with P2 (3.0%) and P3 (3.8%). No significant differences were detected between P2 and P3 and in Farms 2, 3 and 4.

**Fig. 2:**
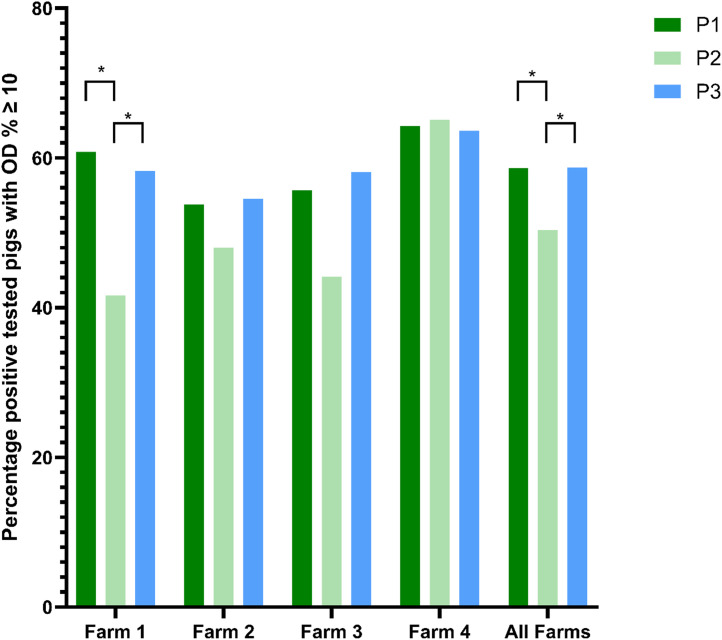
Percentage of positive tested pigs (in absolute terms) on all farms (Farm 1–4 and all together) in three different feeding and vaccination phases with an OD%≥10. Asterisks indicate significant differences between phases within a farm (*p* < 0.05).

Fig. 2 shows the percentage of positive tested pigs on Farm 1 to 4 and all farms together with an OD%≥10 in three different feeding and vaccination phases.

A significant reduction in the proportion of positive cases (OD%≥10) was observed in Farm 1, where prevalence of positive samples decreased from 60.81% in P1 to 41.67% in P2 (*p* < 0.05). However, prevalence of positive samples in P3 (58.27%) was not significantly different from P1 but significantly higher than in P2, indicating a temporary reduction during P2**.** Similarly, no significant temporal differences were observed in the other farms but a trend to the same dynamics as in Farm 1 was noted. When data was pooled across all farms, the proportion of positive samples in P1 (58.7%) and P3 (58.7%) was significantly higher than in P2 (50.3%).

### 3.2 Slaughter findings for farms and feeding time

The prevalence of slaughter findings in various organs and anatomical features (odor, lungs & pleura, heart, ear, liver, and tail) was assessed across six farms during P2 and P3. For each parameter and each farm percentages of positive slaughter findings are reported in Table 2.

**Table 2:** Percentage of slaughter findings (odor, lung and pleura, heart, liver, ear, tail) in pigs on six farms in two different feeding and vaccination phases.

Organ	Farm	P2	P3
Odor	1	1.08^a^	2.46^b^
	2	2.23^a^	1.77^a^
	3	1.89^a^	6.32^b^
	4	2.11^a^	3.13^a^
	5	1.79^a^	3.20^b^
	6	1.79^a^	3.09^b^
Lung and Pleura	1	23.72^a^	16.89^a^
	2	18.78^a^	17.22^a^
	3	19.26^b^	13.55^a^
	4	21.13 ^b^	13.79 ^a^
	5	20.35^a^	30.40^b^
	6	60.31^a^	66.15^b^
Heart	1	2.95^a^	2.27^a^
	2	1.99 ^a^	1.92 ^a^
	3	2.49 ^a^	1.63 ^a^
	4	1.91 ^a^	1.34 ^a^
	5	1.06^a^	2.07^b^
	6	7.51^a^	12.39^b^
Liver	1	0.79 ^a^	0.80 ^a^
	2	0.66 ^a^	0.72 ^a^
	3	0.76^b^	0.25^a^
	4	0.80 ^a^	1.17 ^a^
	5	0.51 ^a^	0.84 ^a^
	6	12.54^b^	2.83^a^
Ear	1	0.20 ^a^	0.00 ^a^
	2	0.08 ^a^	0.19 ^a^
	3	0.45^b^	0.04^a^
	4	0.10 ^a^	0.00 ^a^
	5	0.26 ^a^	0.18 ^a^
	6	0.16 ^a^	0.17 ^a^
Tail	1	1.67 ^a^	1.23 ^a^
	2	1.74 ^a^	1.53 ^a^
	3	1.44 ^a^	1.20 ^a^
	4	1.51 ^a^	0.89 ^a^
	5	0,70 ^a^	1.09 ^a^
	6	0.81 ^a^	0.55 ^a^

a,b Numbers differ significantly between phases (*p* < 0.05).

Significant changes in findings between P2 and P3 were observed in several organs across specific farms. For odor, positive findings increased significantly in Farm 1 (1.08% to 2.46%)**,** Farm 3 (1.89% to 6.32%)**,** Farm 5 (1.79% to 3.20%) and Farm 6 (1.79% to 3.09%) while Farm 2 and 4 showed no significant change. In lungs and pleura, positive rates were significantly higher in P3 compared to P2 for Farm 3, Farm 5, and Farm 6, with the most pronounced increases in Farm 5 (20.35% to 30.40%) and Farm 6 (60.31% to 66.15%). A significant rise in positive findings concerning the heart was observed for Farm 5 (1.06% to 2.07%) and Farm 6 (7.51% to 12.39%), whereas other farms remained stable. In the liver, significant decreases in findings occurred in Farm 3 (0.76% to 0.25%) and Farm 6 (12.54% to 2.83%), with no significant differences in the remaining farms. A slight but significant reduction was detected in positive findings regarding the ears for Farm 3 (0.45% to 0.04%), while all other farms showed no changes. Tail abnormalities did not differ significantly between P2 and P3 for any farm.

Table 3 shows the percentage of pigs tested positive (odor, lung and pleura, heart, liver, ear, tail) on farms in relation to their pathological findings at slaughter in P2 and P3, with Farms 1–4 put together as they underwent adjusted feeding and vaccination, pigs from Farm 5 were vaccinated without adapted feeding and Farm 6 underwent no adjustment.

**Table 3:** Percentage of slaughter findings in pigs on six farms in two different feeding and vaccination phases with Farms 1–4 taken together.

Organ	P3	Farm	P2
Odor	1–4	1.85^Aa^	3.72^Bb^
	5	1.79^Aa^	3.09^Bb^
	6	1.79^Aa^	3.20^Bb^
Lung and Pleura	1–4	20.54^Ab^	15.19^Aa^
	5	60.31^Ba^	66.15^Cb^
	6	20.35^Aa^	30.40^Bb^
Heart	1–4	2.33^Ba^	1.77^Aa^
	5	7.51^Ca^	12.39^Bb^
	6	1.06^Aa^	2.07^Ab^
Liver	1–4	0.75^Aa^	0.68^Aa^
	5	12.54^Bb^	2.83^Ba^
	6	0.51^Aa^	0.84^Aa^
Ear	1–4	0.22^Ab^	0.06^Aa^
	5	0.16^Aa^	0.17^Aa^
	6	0.26^Aa^	0.18^Ba^
Tail	1–4	1.58^Bb^	1.22^Ca^
	5	0.81^Aa^	0.55^Aa^
	6	0.70^Aa^	1.09^Ba^

a,b Numbers differ significantly between phases (*p* < 0.05).

A,B,C Numbers differ significantly between farms (*p* < 0.05).

Across farms, odor-related findings increased significantly from P2 to P3 (e.g., Farm 1–4: 1.85% to 3.72%), while no significant differences were observed between farms within the same phase. For the lungs and pleura**,** Farm 6 consistently showed the highest prevalence (60.31% in P2 and 66.15% in P3), significantly exceeding values observed in Farm 1–4 and Farm 5. Over time, positive findings increased significantly in Farm 5 and Farm 6, whereas Farm 1–4 demonstrated a significant decline. In regard of findings in the heart**,** Farm 6 again exhibited the highest rates (7.51% in P2 and 12.39% in P3), significantly higher than in Farm 1–4 and Farm 5. Both Farm 5 and Farm 6 showed significant increases between phases. Positive findings in the ear were generally low, with Farm 1–4 showing a significant decrease (0.22% to 0.06%) and Farm 5 also displaying a slight but significant reduction, whereas Farm 6 remained unchanged. Regarding the liver**,** Farm 6 demonstrated a markedly high prevalence in P2 (12.54%), which significantly decreased in P3 (2.83%). In contrast, Farm 1–4 and Farm 5 remained stable at very low levels without significant temporal differences. For the tail, no significant changes were observed over time in any farm group. However, differences between farms were evident in both phases, with Farm 1–4 presenting the highest values in P2 and Farm 5 showing a slight but significant increase from 0.70% to 1.09% in P3.

## 4 Discussion

*Salmonella* associated gastrointestinal infections in humans remain a significant public health concern in Germany, across Europe, and globally (EFSA, 2024; RKI, 2020). The European Union has established targets for the control of *Salmonella* and other foodborne zoonoses under Regulation (EC) No. 2160/2003.

This study explored the relationship between rye based, coarse structured feeding, *Salmonella* OD% values, and organ findings observed at slaughter in fattening pigs under practical farm conditions, including the implementation of vaccination against *Lawsonia intracellularis* (LI).

Across the experimental phases, higher inclusion levels of rye were associated with reduced *Salmonella* seroprevalence, particularly in phase P2. This observation is in line with the hypothesis that dietary composition can influence *Salmonella* dynamics under field conditions. In phase P3, where rye inclusion was reduced and LI vaccination was introduced, *Salmonella* prevalence at an OD% threshold of 40 remained low and comparable to P2, without further improvement. At a lower cut off (OD% 10), an increase in seropositive animals was observed. These results indicate that the reduction in *Salmonella* seroprevalence observed with higher rye inclusion was not consistently enhanced under the combined conditions applied in P3, suggesting that the relationship between dietary and immunological interventions may not be additive under all circumstances.

The increase in seroprevalence at lower OD% thresholds in P3 may be associated with immunological responses detectable by ELISA. Vaccination in pigs has been shown to influence antibody responses (e.g., IgA, IgM), which may be detected in serological assays, particularly at low OD values (Theuß et al., 2017). Therefore, a potential influence of LI vaccination on the present ELISA results cannot be excluded (Theuß et al., 2017). At the same time, the use of different OD% cut offs highlights the sensitivity of serological interpretation and suggests that conclusions may vary depending on the diagnostic threshold applied.

The evaluation of post mortem findings revealed an overall low prevalence of pathological lesions across the investigated farms, indicating generally good herd health conditions. However, farm-specific differences were observed. Farms 1–4, which applied rye-based feeding strategies (with or without vaccination), showed stable or decreasing rates of slaughter findings over time. In contrast, Farms 5 and 6, characterized by the absence of dietary intervention and/or vaccination, exhibited higher rates of pathological findings, particularly in P3. These differences suggest that both nutritional management and preventive health strategies may be associated with improved health outcomes at slaughter (Fablet et al., 2018; Fitzgerald et al., 2020).

The limited response observed in farms with initially low seroprevalence suggests a potential threshold effect, where improvements in gut health or pathogen control may no longer translate into measurable changes in serological outcomes. Comparable observations have been reported in previous studies, where intervention effects were more pronounced in herds with higher initial infection pressure (Alban et al., 2012; Roldan-Henao et al., 2023). This highlights the importance of considering baseline herd status when interpreting intervention effects under field conditions.

With regard to explanatory approaches, rye is characterized by a high content of fermentable non starch polysaccharides, which have been associated with effects on gastrointestinal function, digesta viscosity, and microbial fermentation processes (Nielsen et al., 2003; Hankel et al., 2022). These properties may contribute to shifts in the intestinal environment that are less favorable for *Salmonella* persistence (Chuppava et al., 2020; Hankel et al., 2022). However, such mechanisms were not directly assessed in the present study, and conclusions are therefore limited to associations observed under field conditions.

The role of LI vaccination with regard to *Salmonella* prevalence in the present study appeared variable. While vaccination is effective in reducing the burden of proliferative enteropathy and improving intestinal health and performance (Chagas et al., 2026; Kroll et al., 2005; Won et al., 2022), no consistent additive effect on *Salmonella* seroprevalence was observed under the conditions of this study. This observation may be related to the indirect relationship between intestinal integrity and *Salmonella* colonization, as well as to field-specific factors such as timing of vaccination, maternal antibodies, environmental stressors, or concurrent infections (Pluske et al., 2002; Won et al., 2022). In addition, it cannot be excluded that improvements in gut health achieved through dietary measures may have already limited the potential for further measurable effects.

The absence of a clear additive effect of vaccination may also reflect complex interactions between dietary modulation and microbiota dynamics. Previous studies have indicated that combined dietary and immunological interventions may not necessarily result in linear or additive responses (Gioula & Exindari, 2025; Guevarra et al., 2021). However, these interactions were not specifically assessed in the present study.

When compared with previous studies, the observed results differ in part from findings reporting a continuous reduction in *Salmonella* prevalence under comparable interventions (Leite et al., 2018; Meschede et al., 2021). These discrepancies may be related to differences in study design, herd conditions, diagnostic thresholds, or the influence of vaccination on serological measurements (Theuß et al., 2017). In addition, differences in feeding strategies, management practices, and environmental conditions between studies may contribute to variability in outcomes.

Farm specific responses further indicate that the effectiveness of dietary interventions depends on baseline herd conditions. In line with previous studies, stronger effects were observed in farms with higher initial seroprevalence, whereas farms with low baseline prevalence showed limited response (Alban et al., 2012; Jha et al., 2016; Lindberg, 2014; Roldan-Henao et al., 2023).

The generally low prevalence of pathological findings may also reflect favorable herd management conditions, as production related factors such as genetics, housing, and overall herd management are known to influence health outcomes in pig production systems (Fablet et al., 2018; Fitzgerald et al., 2020). Notably, farms without intervention (Farm 6 Table 2 & 3) tended to show less favorable outcomes, whereas farms implementing both interventions showed the most beneficial results, suggesting a potential combined effect. Increased occurrence of olfactory deviations in farms without rye inclusion may be related to differences in dietary composition and associated metabolic processes (Straw et al., 2006).

In summary, the present results indicate that higher inclusion levels of rye in fattening pig diets were associated with lower *Salmonella* seroprevalence under field conditions, while slaughter findings remained generally low across farms. The additional effect of LI vaccination on *Salmonella* outcomes was not consistently evident. Differences between farms highlight the relevance of baseline herd status and management conditions.

Overall, these findings support the role of dietary strategies as part of herd health management in pig production and are consistent with previously reported associations between rye inclusion and reduced *Salmonella* prevalence. At the same time, the results underline that conclusions are limited to field observations, and further controlled studies are required to investigate the underlying biological mechanisms and their interaction with immunoprophylactic measures.

## 5 Conclusions

In conclusion, the present field study indicates that higher dietary inclusion levels of rye in finishing pig diets were associated with reduced *Salmonella* seroprevalence, particularly in herds with higher baseline contamination. In addition, farms applying dietary interventions showed generally lower frequencies of pathological slaughter findings compared to farms without such measures.

The results further suggest that the effectiveness of nutritional and preventive interventions depends on herd-specific conditions, including the initial *Salmonella* status. In herds with low baseline prevalence, no relevant improvements were observed, indicating that intervention effects may be limited under the applied conditions.

The additional vaccination against *Lawsonia intracellularis* (LI) did not result in a consistent further reduction in *Salmonella* seroprevalence under the investigated conditions. While no clear additive effect was observed, the results indicate that the impact of vaccination on *Salmonella* related parameters may vary depending on the specific herd context.

As the present study was conducted under field conditions, the conclusions are limited to observed associations, and no causal relationships can be derived from the current data.

Future research should focus on controlled experimental studies to further investigate the underlying biological mechanisms and to better understand the interaction between dietary composition, intestinal health, and pathogen dynamics. In addition, studies addressing the combined effects of nutritional and immunoprophylactic measures under different conditions would contribute to a more detailed understanding of these interactions.

## Funding

The project was supported by funds of the Federal Ministry of Agriculture, Food and Regional Identity (BMLEH) based on a decision of the Parliament of the Federal Republic of Germany via the Federal Office for Agriculture and Food (BLE) under the innovation support programme (281B101016).

## Data availability

The data used in this study were kindly and voluntarily provided by the participating farms. All datasets used in this study are available from the corresponding author upon reasonable request.

## Generative AI statement

Artificial intelligence was used to support the structuring and formatting of this manuscript. No AI-assisted technologies were used for the generation of scientific content or interpretation of results.

## Ethical approval

All data collection complied with the German Swine *Salmonella* Ordinance and relevant animal welfare legislation. No interventions were performed on live animals; serological testing and organ inspection occurred during the slaughter process.

## CRediT authorship contribution statement

**Jens Gerrit Lindhaus:** Writing – review & editing, Writing – original draft, Validation, Methodology, Investigation, Formal analysis. **Karl Rohn:** Writing – review & editing, Validation, Methodology, Investigation, Formal analysis, Data curation. **Christian Visscher:** Writing – review & editing, Validation, Supervision, Resources, Project administration, Methodology, Funding acquisition, Formal analysis, Data curation, Conceptualization. **Clara Berenike Arnold:** Writing – review & editing, Visualization, Validation, Supervision, Project administration, Methodology, Investigation, Formal analysis, Conceptualization.

## Declaration of competing interest

The authors declare that they have no known competing financial interests or personal relationships that could have appeared to influence the work reported in this paper.
